# Reduced Gene Dosage of the Psychiatric Risk Gene *Cacna1c* Is Associated with Impairments in Hypothalamic–Pituitary–Adrenal Axis Activity in Rats

**DOI:** 10.3390/ijms26125547

**Published:** 2025-06-10

**Authors:** Anna L. Moon, Eleanor R. Mawson, Patricia Gasalla, Lawrence S. Wilkinson, Dominic M. Dwyer, Jeremy Hall, Kerrie L. Thomas

**Affiliations:** 1 Neuroscience and Mental Health Innovation Institute, School of Medicine, Cardiff University, Cardiff CF24 4HQ, UK; anna.moon@hotmail.co.uk (A.L.M.); mawsoner@cardiff.ac.uk (E.R.M.); gasallacantop@cardiff.ac.uk (P.G.); wilkinsonl@cardiff.ac.uk (L.S.W.); hallj10@cardiff.ac.uk (J.H.); 2 Centre for Neuropsychiatric Genetics and Genomics, School of Medicine, Cardiff University, Cardiff CF24 4HQ, UK; 3 School of Psychology, Cardiff University, Cardiff CF10 3AT, UK

**Keywords:** *Cacna1c*, schizophrenia, bipolar disorder, glucocorticoid, anxiety, stress, L-type voltage-gated calcium channel, hippocampus

## Abstract

Common and rare variation in *CACNA1C* gene expression has been consistently associated with neuropsychiatric disorders such as schizophrenia, bipolar disorder, and major depression. However, the underlying biological pathways that cause this association have yet to be fully determined. In this study, we present evidence that rats with a reduced gene dosage of *Cacna1c* have increased basal corticosterone levels in the periphery and reduced the expression of *Nr3c1* encoding the glucocorticoid receptor in the hippocampus and hypothalamus. These results are consistent, with an effect of *Cacna1c* dosage on hypothalamus–pituitary–adrenal (HPA) axis function. Heterozygous *Cacna1c* rats had lower levels of the histone markers H3K4me3 and H3K27acat exon 1_7_ of the *Nr3c1* gene. These histone modifications are typically linked to increased gene expression, but here were not associated with changes in the expression of exon 1_7_ variants under non-stress conditions. Heterozygous *Cacna1c* rats additionally show increased anxiety behaviours. These results support an association of *Cacna1c* heterozygosity with the altered activity of the HPA axis and function in the resting state, and this may be a predisposing mechanism that contributes to the increased risk of psychiatric disorders with stress.

## 1. Introduction

Genetic variation in *CACNA1C*, which encodes the pore-forming subunit of L-type voltage-gated calcium channel (L-VGCCs) Ca_v_1.2, is consistently linked to an increased risk for neuropsychiatric disorders, including schizophrenia and bipolar disorder [1,2,3,4]. Single-nucleotide polymorphisms (SNPs) in intron 3 heighten risk and regulate *CACNA1C* gene expression, including in the brain [5,6,7]. While the effect varies across studies [5,6,8,9], there is evidence that *CACNA1C* risk alleles reduce Ca_v_1.2 expression, particularly in the hippocampus [10]. L-VGCCs are crucial for cell excitability [11] and mediate neuronal excitation–transcription coupling [12]. Hence, reductions in Ca_v_1.2 expression may have significant implications for neuronal, network, and behavioural function relating to risk for neuropsychiatric disorders. Indeed, fine-mapping and eQTL analysis from the largest schizophrenia GWAS to date identified *CACNA1C* as a key candidate contributing to the enrichment of gene sets relevant to synaptic function associated with disease [13].

Stress and dysregulation of the hypothalamic–pituitary–adrenal (HPA) axis are closely linked to psychiatric disorders [14,15,16,17,18]. SNPs in *CACNA1C* interact with stress exposure, influencing the risk for depressive symptoms [19,20] and bipolar disorder [21]. The risk SNP rs1006737 in *CACNA1C* interacts with early-life stress to affect the cortisol-awakening response, a read-out of HPA axis activity [22]. These studies indicate a moderating role of *CACNA1C* variants on the HPA axis and the association between stressors and risk for psychiatric disorders. Preclinical studies also reveal a link between *Cacna1c* and stress responsiveness; mice with altered *Cacna1c* expression show heightened stress susceptibility [23], and a 5-HT neuron-specific knock-out of *Cacna1c* disrupts stress-coping behaviours [24]. In addition, animal studies show that Ca_v_1.2 expression is highly responsive to corticosteroids and stress [25,26,27,28,29,30,31]. Thus, the interplay of Ca_v_1.2 and the HPA axis may represent a mechanism for risk of developing neuropsychiatric disorders.

When a stressor is encountered, the HPA axis triggers the release of glucocorticoids—cortisol in humans or corticosterone in rodents [32]. Glucocorticoids regulate various functions in the body, including metabolism and cognition, through binding to low-affinity glucocorticoid receptors (GRs) or high-affinity mineralocorticoid receptors (MRs) in peripheral tissue and the brain, including the hippocampus, hypothalamus, and pituitary, through both membrane-bound nongenomic mechanisms to control neuronal activity and nuclear transcriptional activity to regulate gene expression [33]. Central GRs also initiate negative feedback to reduce further cortisol production, ending the acute stress response [34,35,36,37]. MRs, which are primarily active under basal conditions, inhibit the HPA axis, regulating system reactivity [38,39,40]. This system provides adaptive advantages under brief stress, promoting survival via allostatic regulation. However, chronic stress can render the system maladaptive, changing basal cortisol levels. Hypercortisolaemia, with reduced glucocorticoid feedback, is linked to depression, anxiety, and other disorders [41,42]. Conversely, hypocortisolaemia and high-stress sensitivity are tied to traumatic or chronic stress exposure, particularly early-life or childhood trauma experiences and conditions like post-traumatic stress disorder (PTSD), atypical depression, and chronic fatigue syndrome [43,44,45].

This study sought to directly investigate how variation in *Cacna1c* dosage impacts the HPA axis using a heterozygous *Cacna1c* rat model that reflects the reduced hippocampal *CACNA1C* expression associated with psychiatric disease. Given the evidence of a relationship between activation of the HPA axis and Ca_v_1.2 expression and function after stress, we hypothesised that variation in the dosage of *Cacna1c* would impact the HPA axis. Here, we investigated whether *Cacna1c* haploinsufficiency in rats was associated with altered central glucocorticoid receptors and circulating corticosterone levels under baseline conditions. This explicit aim is an important first step in disambiguating the mechanism by which altered Ca_v_1.2 signalling impacts the HPA axis under stress and for a better understanding of the causal mechanisms through which genetic variation in *CACNA1C* might contribute to risk for psychiatric disorders in humans. Briefly, we sought to determine whether reduced *Cacna1c* expression resulted in a dysregulated HPA axis prior to stress.

## 2. Results

### 2.1. Cacna1c Heterozygosity Is Associated with Decreased GR Expression in the Hypothalamus and Hippocampus

**Figure 1:**
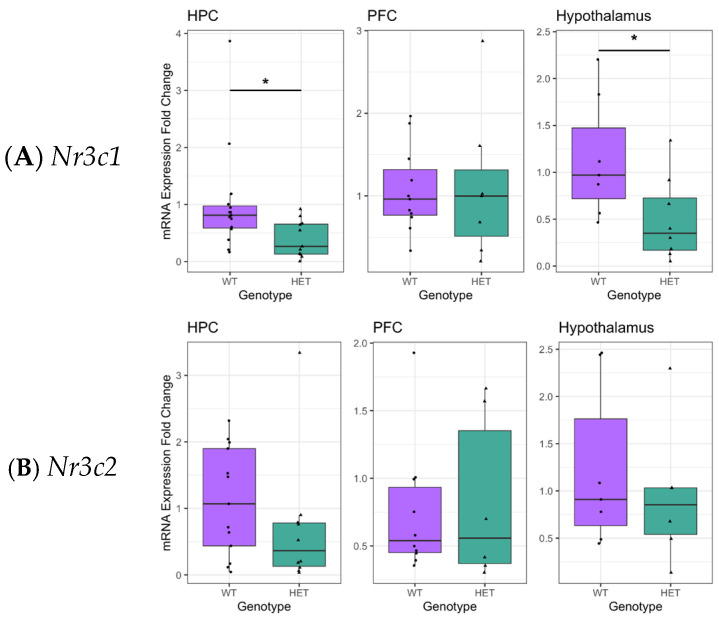
(**A**). *Cacna1c*^+/−^ rats (HET) had reduced *Nr3c1* gene expression in the hippocampus and the hypothalamus compared to wild types (WTs). (**B**). There were no differences in *Nr3c2* expression in any brain region tested. Medians and quartiles are depicted on each plot (whiskers run to the smallest datapoint within 1.5 × IQR below Q1 and the largest datapoint within 1.5IQR above Q3). Measures from individual rats are shown as black dots. Results were considered significant if *p* < 0.05 (*). Although transformations were used in the analysis, for clarity, untransformed data are presented here (see Figure S1 for visualisation of transformed data). *Nr3c1*: hippocampus, HET n = 11, WT n = 15; PFC, HET n = 7, WT n = 11; Hypo HET n = 8, WT n = 7. *Nr3c2*: hippocampus, HET n = 10, WT n = 13; PFC, HET n = 6, WT n = 10; Hypo HET n = 6, WT n = 7.

*Nr3c1* and *Nr3c2* encode GRs and MRs, respectively, and play a key role in the HPA stress response network. To assess the effect of reduced *Cacna1c* gene dosage on HPA functioning, brain *Nr3c1* and *Nr3c2* expression in the *Cacna1c*^+/−^ model was measured. In the hippocampus, a stress-sensitive region with a high proportion of GRs and MRs, a reduction in *Nr3c1* expression was seen in *Cacna1c*^+/−^ rats (*t*_(24)_ = −2.654, *p* = 0.014; sqrt-transformed) and similarly in the hypothalamus (*t*_(13)_ = −2.486, *p* = 0.027; sqrt-transformed), whereas in the prefrontal cortex (PFC), no differences were measured compared to WTs (*t*_(21)_ = −0.504, *p* = 0.621; log-transformed) (Figure 1A). No difference was observed in *Nr3c2* expression in any region investigated (hippocampus: *t*_(21)_ = −1.430, *p* = 0.168; (log-transformed), hypothalamus: *t*_(11)_ = −0.805, *p* = 0.438 (log-transformed), PFC: *t*_(14)_ = 0.005, *p* = 0.944 (inverse-square-transformed)) (Figure 1B). Transformations to the dependent variables were used as indicated to ensure the linear regression models adhered to assumptions of normality and homogeneity of the variance of residuals. Different transformations were required in different cases, and the appropriate transformation was determined using Box–Cox analysis (see Section 4.7). A selective decrease in *Nr3c1* expression in the hippocampus and hypothalamus was seen in two separate cohorts of male *Cacna1c*^+/−^ and WTs and therefore, data were combined (Figure S3). The reproducibility of this finding highlights the robustness of the effect of *Cacna1c* gene dose on *Nr3c1* expression in these brain regions.

### 2.2. Epigenetic Changes in exon1_7_ of Nr3c1 in the Hippocampus of Heterozygous Cacan1c Rats

#### 2.2.1. DNA Methylation

**Figure 2:**
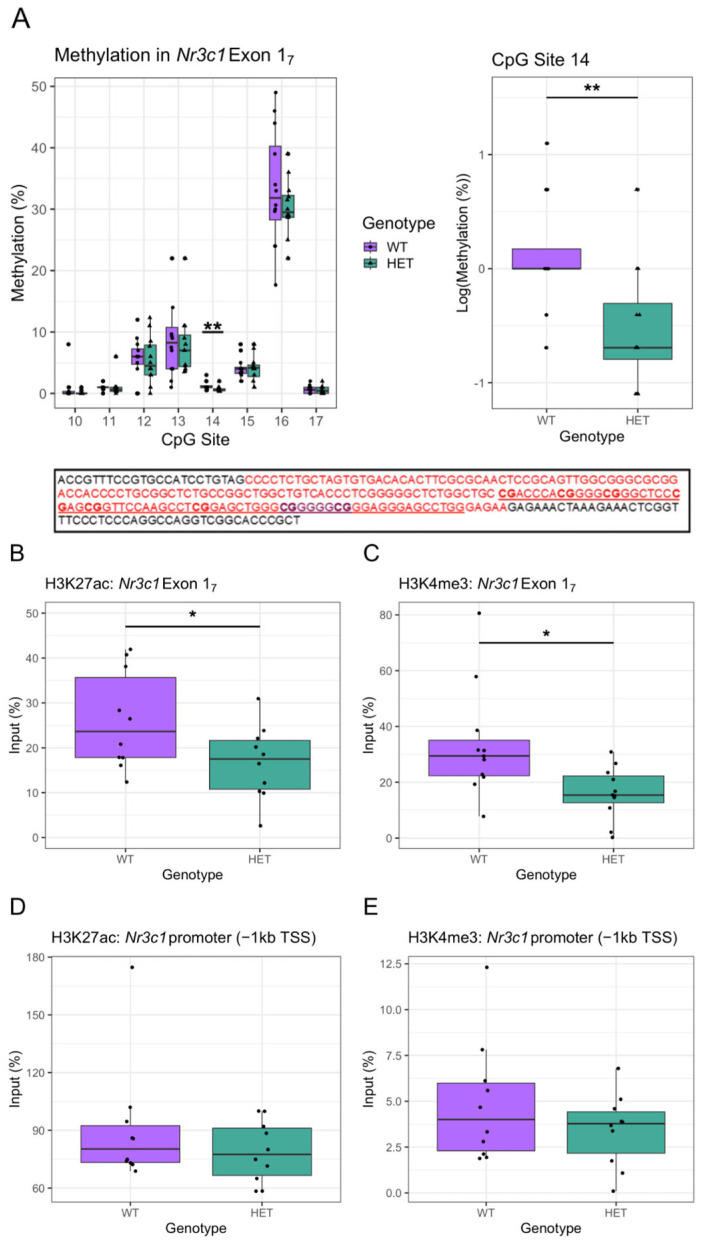
(**A**) *Top*: The % of methylation at each targeted CpG site in the hippocampus of WT rats and *Cacna1c*^+/−^ rats is displayed (*top left*) with no significant differences between the genotypes, with the exception of CpG 14 (*top right*). The highest % methylation was within at the 5′ CpG site (CpG_16_) within the NGFIA binding site. *Bottom*: Schematic showing the genetic sequence that indicates Exon 1_7_ within the promoter region of *Nr3c1* in the rat [49]. The red region indicates the sequence of Exon 1_7_, with the NGFIA binding site contained within, highlighted in purple. The sequence analysed by this study is underlined, and the CpG dinucleotides investigated are in bold (corresponding to CpGs 10–17 [49]. n = 12 per group. (**B**–**E**) *Cacna1c*^+/−^ rats show reduced DNA interacting with histone modification markers of active transcription H3K4me3 and H3K27ac within the exon 1_7_ region (**B**,**C**). No differences were seen in the region closer to the transcription start site (**D**,**E**). Medians and quartiles are depicted on each plot (whiskers run to the smallest datapoint within 1.5 × IQR below Q1 and the largest datapoint within 1.5IQR above Q3). n = 10 per group except c, where n = 11. Measures from individual rats are shown as black dots. Although transformations were used in the analysis, for clarity, untransformed data are presented here (see Figure S2 for visualisation of transformed data). Results were considered significant if *p* < 0.05 (*) or *p* < 0.05 (**).

The decrease in *Nr3c1* expression in *Cacna1c*^+/−^ rats may be due to epigenetic effects. The *NR3C1* gene contains alternative non-coding exon 1 variants that contain sites of epigenetic modification that regulate *NR3C1* gene transcription. Focus has been on exon 1_7_ in rat hippocampi, which have been demonstrated to have an epigenome that is susceptible to environmental influences such as early-life maternal care, resulting in changes in DNA methylation at CpG site 16 containing the 5′ binding site for NGFIA and altered hippocampal GR levels [46,47]. Furthermore, in humans, DNA methylation in the analogous 1F CpG cluster region has been associated with psychopathology [48]. We explored DNA methylation in this region, including the CgG site 16 within exon 1_7_. In the hippocampus we found no differences in methylation of any of the investigated CpG sites between the male *Cacna1c*^+/−^ and WTs, with the exception of decreased methylation in the poorly methylated CpG site 14 in *Cacna1c*^+/−^ rats (*t*_(22)_ = −3.006, *p* = 0.007; log-transformed) (Figure 2A, Table S1).

#### 2.2.2. Histone Modifications

As there was no change in direct DNA methylation within seven of the eight targeted CpG sites in exon 1_7_, we utilised ChIP-qPCR to investigate histone modifications that correlate with the expression of *Nr3c1* in the hippocampus of hemizygous rats. Histone modifications in the exon 1_7_ GR promotor region associated with altered GR expression in the hippocampus have been reported [46,47,50]. Acetylation of H3K27 and tri-methylation of H3K4 are markers of active gene transcription [51,52]. Using primers to target *Nr3c1* exon 1_7_, a reduction in H3K27ac (*t*_(18)_ = −2.169, *p* = 0.044) (Figure 2B) and H3K4me3 (*t*_(20)_ = −2.722, *p* = 0.013; sqrt-transformed) interaction within the *Nr3c1* exon 1_7_ region was seen in *Cacna1c*^+/−^ compared to WT rats (Figure 2C). There were no genotype differences in DNA interacting with H3K27ac (*t*_(18)_ = 1.111, *p* = 0.281; inverse-transformed) (Figure 2D) or H3K4me3 (*t*_(19)_ = −1.190, *p* = 0.249; sqrt-transformed) (Figure 2E) within a region −1 kb upstream of the TSS of the *Nr3c1* promoter region. These results suggest that reduced *Nr3c1* expression in the hippocampus of *Cacna1c*^+/−^ rats could be at least partly driven by altered histone modification within the promoter of *Nr3c1* within exon 1_7._ Despite a degree of spatial selectivity in altered H3K27ac or H3K4me3 levels in the GR promotor, there was no difference in mRNA expression of exon 1_7_ itself (Figure S3) in *Cacna1c*^+/−^ rats.

### 2.3. Cacna1c Heterozygosity Increases Peripheral Corticosterone Levels

**Figure 3:**
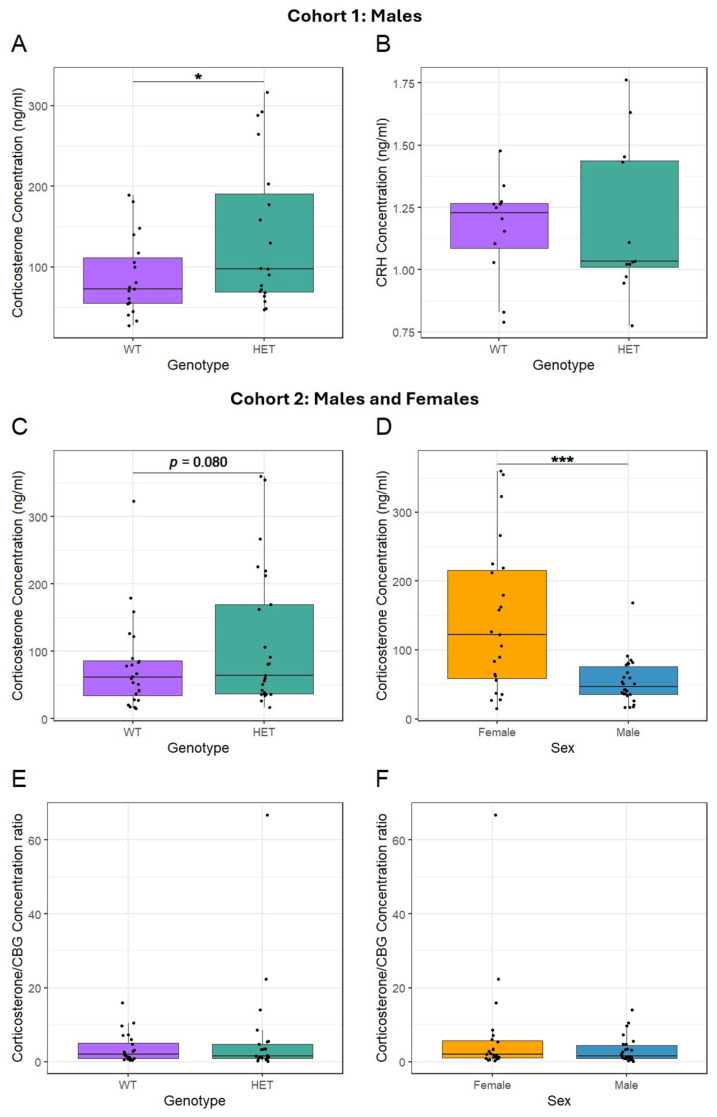
(**A**) Male *Cacna1c*^+/−^ rats (HETs) had higher circulating corticosterone levels than WT males (n = 19 per group). (**B**) Male *Cacna1c*^+/−^ rats had similar CRH hormone levels to WT males (n = 12 per group). In a separate mixed-sex cohort (n: male WT = 13, male HET = 13, female WT = 11, and female HET = 12) (**C**), *Cacna1c*^+/−^ rats showed an increased peripheral corticosterone concentration compared to WT rats. (**D**) A profound sex difference in corticosterone levels was also observed, with females showing higher levels than males. (**E**) There was no difference in the ratio of peripheral corticosterone/CBG between genotypes (*p* = 0.248). (**F**) Corticosterone/CBG did not differ between sexes (*p* = 0.199). There were no sex-by-genotype interactions (*p* > 0.05, see main text for details), and main effects are presented only. Medians and quartiles are depicted on each plot (whiskers run to the smallest datapoint within 1.5 × IQR below Q1 and the largest datapoint within 1.5IQR above Q3). Measures from individual rats are shown as black dots. Although transformations were used in the analysis, for clarity, untransformed data are presented here (see Figure S4 for visualisation of transformed data). * *p* < 0.05, *** *p* < 0.001.

Corticosterone and corticotrophin-releasing hormone (CRH) concentrations were measured in blood plasma in an initial all-male cohort of *Cacna1c*^+/−^ rats. There was an increase in peripheral corticosterone in *Cacna1c*^+/−^ rats (*t*_(36)_ = 2.078, *p* = 0.045; log-transformed) (Figure 3A) but no difference in peripheral CRH (*t*_(22)_ = 0.011, *p* = 0.991; log-transformed) (Figure 3B).

Given the observation of a genotype effect on corticosterone levels in males and the known sex differences in glucocorticoid biology [53], investigations were expanded to include sex as a variable. To assess whether the genotype difference reflected bioavailable corticosterone levels, and to determine whether the effect was found in both sexes, corticosterone and corticosteroid-binding globulin (CBG) were subsequently measured in a mixed-sex cohort. For corticosterone levels a sex-by-genotype interaction was not significant (*t*_(46)_ = −0.950, *p* = 0.347; log-transformed), and moreover, the AIC score was improved by removing this term from the regression model. Consequently, all results reported do not include the sex-by-genotype interaction in the model. There was a marginal difference in plasma corticosterone levels between the *Cacna1c*^+/−^ and WT rats in the mixed-sex cohort (*t*_(47)_ = 1.427, *p* = 0.080, one-tailed and log-transformed, Figure 3C). Females were shown to have higher corticosterone levels than males (*t*_(47)_ = 3.749, *p* < 0.001; log-transformed) (Figure 3D).

When corticosterone travels through the blood, it is bound to CBG, which provides protection from degradation, but when bound to CBG, corticosterone is also unable to act on target tissues [54]. Hence, assessing whether there are differences in the ratio of corticosterone to CBG is important to better understand how changes in corticosterone might impact upon HPA axis targets. Higher and lower corticosterone-to-CBG ratios would indicate increased or decreased bioavailable corticosterone, respectively. However, there was no difference in corticosterone/CBG ratio between genotypes (*t*_(46)_ = −0.688, *p* = 0.248; one-tailed) (Figure 3E). Similarly, there was no difference in the corticosterone/CBG ratio between sexes (*t*_(46)_ = −1.304, *p* = 0.199) (Figure 3F). No interaction was observed between sex and genotype (*t*_(46)_ = 0.980, *p* = 0.332). Together these data show that there was evidence of an increase in circulating corticosterone levels in *Cacna1c*^+/−^ rats under baseline non-stressed conditions, which was most evident in males. There were also higher levels of corticosterone in females compared to males. These increases in corticosterone levels were not accompanied by a compensatory change in the bioavailability of corticosterone.

### 2.4. Increased Anxiety-Associated Behavioural Responses in Cacna1c Heterozygous Rats

To determine whether the elevated circulating corticosterone levels we observed in the *Cacna1c*^+/−^ rats was correlated with increased anxiety like behaviours [55], we assessed emotional reactivity as the time spent in the central zone of an open field (OF) and in the open arms of the elevated plus maze (EPM).

**Figure 4:**
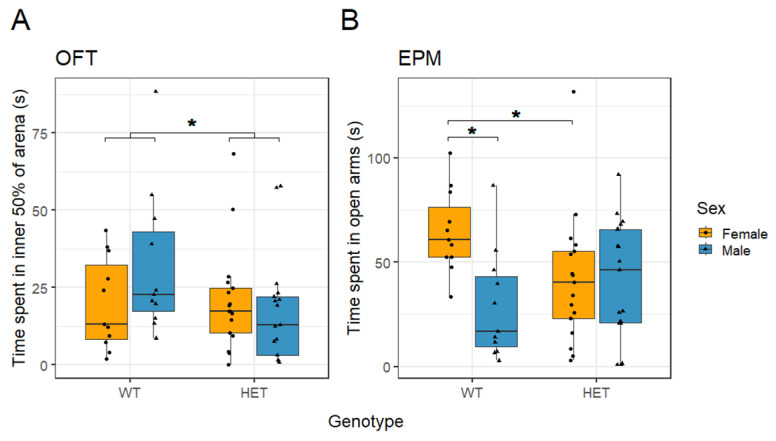
(**A**) There was a reduction in time spent in the inner 50% of an OF arena in *Cacna1c^+/^-* rats (HET) compared to WT animals. (**B**) In the same cohort, there were no differences of genotype regarding the time spent in the open arms of the EPM. However, female rats spent more time in the open arms compared to males, which was associated with WT females spending more time in the open arms than WT males. Medians and quartiles are depicted on each plot (whiskers run to the smallest datapoint within 1.5 × IQR below Q1 and the largest datapoint within 1.5IQR above Q3). Although transformations were used in the analysis, for clarity, untransformed data are presented here (see Figure S6 for visualisation of transformed data). Measures from individual rats are shown as black dots. * *p* < 0.05. Male HET n = 17; male WT n = 11; female HET n = 17; female WT n = 11.

*Cacna1c*^+/−^ rats spent less time in the inner 50% of the OF arena than WT rats (*t*_(52)_ = −2.277, *p* = 0.027; sqrt-transformed, Figure 4A). There was no difference between the sexes (*t*_(52)_ = −1.509, *p* = 0.137; sqrt-transformed) and no interaction between sex and genotype (*t*_(52)_ = 1.663, *p* = 0.102; sqrt-transformed) for the time spent in the inner 50% zone of the OF. There were no differences in locomotor activity between the genotypes or the sexes analysed as total distance travelled (Figure S5A,C).

In the EPM there was no difference in the time spent in the open arms between *Cacna1c* genotypes (*t*_(52)_ = 1.043, *p* = 0.302; sqrt-transformed, Figure 4B). However, females spent significantly more time in the open arms compared with males (*t*_(52)_ = 3.068, *p* = 0.003; sqrt-transformed). This sex difference was found to interact with genotype (*t*_(52)_ = −2.257, *p* = 0.028; sqrt-transformed), as reflected in female WT rats spending more time in the open arms compared with male WT rats (*t*_(20)_ = 3.826, *p* = 0.001; sqrt-transformed), and this sex difference was ameliorated in the *Cacna1c*^+/−^ rats (*t*_(32)_ = 0.193, *p* = 0.848; sqrt-transformed). When stratified by sex, WT females spent more time in the open arms compared with HET females (*t*_(26)_ = −2.457, *p* = 0.021; sqrt-transformed), while no differences were observed between genotypes in males (*t*_(26)_ = 0.938, *p* = 0.357; sqrt-transformed). The overall sex difference in the EPM was concomitant with females showing increased (*t*_(52)_ = −2.042, *p* = 0.046; inverse-transformed) locomotor activity compared to males (Figures S5B and S6B). Thus, *Cacna1c*^+/−^ rats showed evidence of increased anxiety in two tests. In the OFT, they spent less time in the central zone. By contrast, only female *Cacna1c*^+/−^ rats spent less time in the open arms of the EPM regardless of females being less anxious and more active than males in this task.

## 3. Discussion

This study assessed how reduced *Cacna1c* gene dosage affects HPA axis function using a heterozygous *Cacna1c* rat (HET) model. We found decreased *Nr3c1* mRNA encoding GRs in the hippocampus and hypothalamus of *Cacna1c*^+/−^ rats and lower H3K4me3 and H3K27ac histone modifications linked to gene activation in exon 1_7_ of *Nr3c1*. The selective decrease in GR, but not MR, expression in the hippocampus and hypothalamus, combined with evidence of increased plasma corticosterone levels and unchanged CRH levels under baseline conditions, suggests GR resistance and altered HPA feedback in the HETs [33]. Thus, *Cacna1c* knockdown leads to the development of an HPA axis that may function abnormally. Evidence for increased innate anxiety responses were also observed in the *Cacna1c*^+/−^ rat model.

We observed reduced *Nr3c1* gene expression in the hippocampus and hypothalamic regions of *Cacna1c*^+/−^ rats, but not in the PFC, indicating regional selectivity of *Cacna1c* hemizygosity on HPA axis function. As such it is interesting to note that GR activity in the hippocampus and PVN is crucial for negative feedback and adaptive regulation of the HPA axis after stress [33]. DNA methylation, particularly at CpG site 16 of *Nr3c1,* inversely affects *NGFIA* transactivation of exon 1_7_-containing GR transcripts in the hippocampus [52,55]. We found no altered DNA methylation at CpG_16_ in *Cacna1c*^+/−^ rats, explaining the unchanged expression of exon 1_7_-containing GR transcripts. However, we observed epigenetic changes in exon 1_7_ of *Nr3c1*, including decreased CpG_14_ methylation and lower H3K4me3 and H3K27ac, which are linked to transcriptional activation and repression. While epigenetic changes within *Nr3c1* are associated with *Cacna1c* hemizygosity, the full epigenetic signature that results in reduced GR expression in the hypothalamus and hippocampus associated with *Cacna1c* haploinsufficiency remains to be determined.

DNA demethylation at *Nr3c1* CpG_16_ and active histone marks like H3K27ac at exon 1_7_ are positively correlated with GR expression and negatively correlated with depressive-like behaviour [46,47,50]. Notably, this dynamic relationship has been observed in studies following early-life stress. Our findings of unchanged CpG_16_ exon 1_7_ methylation and exon 1_7_ transcript expression under basal conditions in *Cacna1c*^+/−^ rats are concordant with CpG_16_ methylation at exon 1_7_ of *Nr3c1* being mechanistically linked to stress-associated functional adaptations and psychopathy [48]. However, histone modifications in exon 1_7_ of *Cacna1c*^+/−^ rats suggest this region is susceptible to epigenetic changes due to environmental stress and/or genetic factors. In the absence of a measurable change in the expression of exon 1_7_ variants, the decrease in *Nr3c1* expression measured in the stress-naive *Cacna1c*^+/−^ rats largely relates to a decrease in the expression of splice variants not containing exon1_7_. Nevertheless, the epigenetic changes in exon1_7_ associated with *Cacna1c* heterozygosity may confer an altered vulnerability to stress to impact HPA axis regulation, and this remains to be determined in future studies.

The allosteric load model suggests acute stress can be beneficial by triggering adaptive coping mechanisms, but chronic, excessive, or uncontrollable stress leads to a transition to a maladaptive state [56]. In this model, glucocorticoids and their receptors are crucial, typically showing a U-shaped function: both high and low levels suppress or fail to engage synaptic and neuroplasticity mechanisms needed for adaptation [57]. Chronic hyperactivation of the HPA axis, with elevated cortisol/corticosterone (CORT) and decreased GR feedback, may eventually lead to a self-preserving downregulation of the HPA axis and reduced CORT production [58]. Factors affecting this adaptive-to-maladaptive transition include the duration and intensity of HPA activation and the timing of stress exposure [58]. Thus, the hypercortisolism and GR resistance in *Cacna1c*^+/−^ rats may represent a model of heightened vulnerability to stress and stress-associated cognitive, behavioural, metabolic, and neuroimmune changes linked to various psychiatric disorders.

*Cacna1c*^+/−^ rats exhibit evidence of increased anxiety-like behaviours compared to WT rats. These findings align with observations in haploinsufficient mice [59,60]. The anxiety-like behaviour changes are not confounded by genotype effects on motor behaviour, consistent with data from haploinsufficient male mice [31,59]. In the OF, *Cacna1c*^+/−^ rats showed more anxiety-like behaviour, where they spent less time in the open central region of the arena. In the EPM test, only female *Cacna1c*^+/−^ rats showed increased anxiety, spending less time in the open arms. These observations may reflect that the OF and EPM tasks differentially engage motivational components of exploration (fear vs. curiosity, which together mediate risk taking in open spaces), in so much as the OF is forced exploration of an open environment [61]. Our data, along with previous studies [60,61], indicate that females may be more sensitive to Ca_v_1.2 reduction’s effects on anxiety-like behaviour, reflecting sex-specific differences in motivational processes. This sensitivity in *Cacna1c*^+/−^ rodents parallels the observation that women with *CACNA1C* risk variants are more prone to mood disorders and anxiety than men [60,62]. Note that in these experiments, rats spent relatively low levels of time in the central or open arms of the OF and EPM, respectively, thus demonstrating high levels of anxiety even in the WT group. In addition, the measurement of low levels of dwell time in the central or open arms potentially reflect non-optimal estimations of anxiety differences between experimental groups due to the potential floor effect on recording values in this range. Future studies using optimised experimental conditions for OF and EPM will give a more accurate estimate of the magnitude of the change in anxiety in the *Cacna1c*^+/−^ rats. Notwithstanding, our data are concordant with previous studies.

Reports of altered anxiety-like behaviour in rodent models of *Cacna1c* deletion can vary depending on the targeted cell type or brain regions. For instance, global *Cacna1c* haploinsufficiency models and those with conditional knockout in excitatory neurons, especially in the PFC [63] or D1R-expressing neurons [31], show increased anxiety-like behaviours. In contrast, models with Ca_v_1.2cKO in both excitatory and inhibitory neurons exhibit normal anxiety-like behaviour [64,65]. Cell-restrictive *Cacna1c* deletion models help identify the locus of anxiety dysregulation and other functional impacts of altered Ca_v_1.2 levels, as well as underlying neural mechanisms, such as the E/I balance. This understanding is crucial for developing targeted therapies based on using arguably more disease-relevant *Cacna1c* haploinsufficiency models, which affect Ca_v_1.2 levels in both glial and neuronal populations in a regionally selective manner [66,67].

Increased plasma corticosterone levels in *Cacna1c*^+/−^ rats may contribute to their heightened anxiety response, as corticosterone treatment and HPA activation are linked to anxiety-like behaviours in rodents [55,67,68,69,70,71]. The decreased GR expression in the hippocampus and hypothalamus may also play a role. Reduced GR expression leads to less negative feedback to the HPA, increasing circulating corticosterone. Additionally, deletion of GRs in forebrain excitatory cells, including the hippocampus but not the PVN, is associated with increased anxiety-like behaviour and hypercortisolism [72]. We also observed no changes in MR expression in the hippocampus, which typically plays a permissive role in corticosterone regulation of anxiety [73]. These findings suggest that the elevated MR/GR ratio in the hippocampus and possibly other regions of the neural circuits regulating anxiety could underlie the increased innate anxiety in *Cacna1c*^+/−^ rats.

We observed higher corticosterone levels in females, consistent with previous findings [74,75]. Although both sexes show similar diurnal patterns of baseline corticosterone [76], early-morning nadir corticosterone levels are higher in female rodents with reduced GR levels in the PVN [77]. The sex differences in circulating corticosterone we measured during the first few hours of the morning may have its origins in an altered sensitivity of HPA feedback in *Cacna1c*^+/−^ females carrying reduced hypothalamic GR. Regardless of the exact cause, higher corticosterone levels in females suggest a possible difference in anxiety-related behaviours, as corticosterone is strongly linked to anxiety—at least in males. CBG, which limits corticosterone bioavailability and regulates corticosterone release and emotional reactivity, is influenced by gonadal oestrogen [78]. The oestrous-mediated control of CBG and therefore bioavailable corticosterone in females may explain why similar levels of anxiety are seen in both sexes. We did not control for the stage of oestrous here, and the association between bioavailable corticosterone and anxiety in females remains to be investigated. The effect of genotype on corticosterone levels seen in males but not in a mixed-sex cohort may have its origin in the higher variance in this measure we observed in females, which may be related to fluctuations in gonadal hormones that regulate the HPA axis, [79], for review). Future studies should consider oestrous stage to better understand sex differences in bioavailable corticosterone and anxiety behaviour in *Cacna1c*^+/−^ rats.

Recent studies suggest that Ca_v_1.2 plays a role in how the human HPA system adapts to stress [22,80]. Both *CACNA1C* gene variants and their methylation state influenced the body’s natural rise in cortisol upon waking, which is considered a positive adaptive stress response, with non-psychiatric-risk allele carriers who had experienced early-life stress showing an increased cortisol awaking response and adaption. The exact mechanism behind this is unclear, but one theory suggests that stress and corticosterone trigger more corticosterone release through a positive feedback loop, which increases intracellular calcium and Ca_v_1.2 expression in the hippocampus (see [81], for review). Our previous investigations have shown reduced intracellular calcium signalling and impaired LTP in the hippocampus of *Cacna1c*^+/−^ rats [82]. Together these data suggest a role for Ca_v_1.2 in the calcium signalling plasticity mechanisms associated with CORT-dependent HPA adaptation [83]. As such, diminished Ca_v_1.2 expression and Ca_v_1.2-dependent calcium signalling result in a dysregulated HPA axis, characterised by reduced negative feedback, and predict a maladaptive response to stress via impaired synaptic plasticity. Ca_v_1.2-associated alterations in HPA originate developmentally, with evidence from neuron-specific *Cacna1c*^−/−^ mice that spontaneous calcium activity is perturbed from early embryogenesis, which is associated with altered brain structure (including the hippocampus) and anxiety in adulthood [71], and that embryonic, but not adult-induced, *Cacna1c* depletion results in impaired hippocampal function, synaptic plasticity and anxiety coupled with an increased vulnerability to the effects of stress [19].

A limitation of this study is that not all measures of basal HPA-related activity and function investigated were made in both male and female rats. We acknowledge that investigations in both sexes in animals are required to understand the biological basis in differences in vulnerability to stress-associated psychiatric disease [84]. We show evidence of a dysregulation of the HPA axis in unstressed males with reduced dosage of *Cacna1c*, a gene well established to be linked to an increased risk for neuropsychiatric disorders, and an associated anxiety phenotype. Our more preliminary data in unstressed females are broadly concordant with those in males despite evidence of differences in the underlying biology (higher peripheral corticosterone levels). This indicates the importance of sex as a biological variable if studies are not only going to illuminate the source of sex differences in vulnerability to stress but also the interaction between stress and genetic risk for disease.

Our data indicate that the psychiatric risk rat model of *Cacna1c* haploinsufficiency shows an altered HPA axis in adulthood, with evidence of increased baseline plasma corticosterone levels, reduced GR expression (and an elevated MR/GR ratio) in the hypothalamus and hippocampus, and heightened anxiety-like behaviour. This phenotype suggests the model has increased vulnerability to stress [83]. Future studies should investigate the behavioural and physiological effects of stress in this model, especially following early-life stress, which is linked to altered HPA axis function and psychiatric illness [45,80,81]. It is crucial to include both sexes in these studies due to sex differences in HPA axis development and regulation [83], which is important for developing treatments for psychiatric disorders in both men and women [85].

## 4. Materials and Methods

### 4.1. Animals

*Cacna1c* heterozygous rats (*Cacna1c*^+/−^) on a Sprague Dawley background (TGR16930, Horizon, Sage Research Labs, Boyertown, PA, USA) and wild-type littermates were bred in-house and housed in mixed-genotype groups of 2–4. The *Cacna1c*^+/−^ model has a 40–50% decrease in *Cacna1c* mRNA and protein levels in the brain [66]. Animals were housed under a 12:12 h light–dark cycle with ad libitum access to food and water. Experiments were conducted in accordance with the UK Animals (Scientific Procedures) Act 1986.

All tissue samples were taken for analysis between 10.00 and 14.00 and rapidly frozen and stored at −80 °C prior to assay. Behavioural experiments were conducted between 10.00 and 14.00. Peripheral hormone levels, and gene expression and epigenetic modifications measured in individual bilateral samples of medial PFC, whole hippocampus (dorsal plus ventral), and hypothalamus as indicated were measured in an all-male cohort (WT = 38 *Cacna1c*^+/−^ = 34). Peripheral hormone levels (Figure 3A,B) were measured in a random selection of 19 samples per genotype, with CRH measures nested (WT = 12, *Cacna1c*^+/−^ = 12). Sample preparation requirements necessitated the analysis of brain tissue in separate samples: rt-qPCR (Figure 1) WT = 15, *Cacna1c*^+/−^ = 11; pyrosequencing (Figure 2A,B) WT n = 12, *Cacna1c*^+/−^ = 12; histone modifications (Figure 2B–E) WT = 11, *Cacna1c*^+/−^ = 11. Where the final n quoted in the figure legends differs from the animal number per experiment, this is due to sample attrition, and all available measures were included for analysis. Subsequent hormone expression assessments (Figure 3C–F, WT = 24, *Cacna1c*^+/−^ = 25) and behavioural experiments (Figure 4, WT = 22, *Cacna1c*^+/−^ = 34) were conducted on separate mixed-sex cohorts to investigate. Stage of oestrous in female rats was not measured.

### 4.2. ELISA

Trunk blood samples were collected into 2 mL lithium heparin Vacuette tubes (Grenier Bio-One, Kremsmünster, Austria) between 10:00 a.m. and 14:00 p.m., being randomised across groups. Plasma was extracted via centrifugation (4000 rpm for 10 min at 4 °C) and corticosterone measured using the Abcam CORT ELISA Kit (ab10882, Cambridge, UK) using a 1:100 plasma dilution. CRH was analysed using the CRH ELISA Kit (ABIN2871385, Antibodies Online, Aachen, Germany).

### 4.3. RT-qPCR

RNA was extracted from tissue (20–30 mg tissue per sample) using the RNeasy Kit (Qiagen, Manchester, UK), following the manufacturer’s instructions. DNAse was treated (Ambion TURBO DNA-free Kit, Austin, TX, USA) and converted to cDNA (cDNA Easy Premix (Random Hexamers) Clontech, Mountain View, CAL, USA) at 42 °C for 75 min and then 80 °C for 15 min. Then, 15 μL qPCR reactions (1:25 primers (10 μM), 1:3 cDNA (5 ng/μL), and 1:2 SYBR-Green SensiMix (Bioline, Toronto, ON, Canada) were run at 95 °C for 10 min, followed by 45 cycles of 95 °C (15 s), 60 °C (60 s), 55 °C (60 s), and 95 °C (15 s). *Gadph* and *Hprt* were used as housekeeping controls. Primer sequences: *Nr3c1* (encoding GR) *F:* 5′-AGCACAATTACCTTTGTGCTGGA, R: 5′-TTCGATAGCGGCATGCTGGA; *Nr3c2* (encoding MR) F: 5′-AATAACGTCCCTCTGCGCTC, R: 5′-GCCTGAAGTGGCATAGCTGA; *Gapdh* F: 5′-TCTCTGCTCCTCCCTGTTCT, R: 5′-TACGGCCAAATCCGTTCACA. *Hprt*: F: 5′-TCCTCCTCAGACCGCTTTTC, R: 5′-ATCACTAATCACGACGCTGGG. Quantification was carried out using the comparative Ct method (2^−ΔΔCT^).

### 4.4. Pyrosequencing

Genomic DNA was extracted from hippocampal tissue samples using the DNeasy Blood and Tissue Kit (Qiagen). Unmethylated cytosines in DNA samples (0.05–100 ng/μL) were sodium bisulphite converted to uracil using the EpiTect Sulphite Kit (Qiagen). Primers were designed to target *Nr3c1* exon 1_7_ using Qiagen PyroMark 2.0 Assay Design software F: 5′-TTGTTATTTTAGGGGGTTTTGGTT, R: 5′-[Biotin] AAAAAAACCCAATTTCTTT-AATTTCTCTTC. Bisulphite-converted DNA was subject to two rounds of PCR (PyroMark PCR Kit, Qiagen) to obtain the amplicon of interest. The biotinylated PCR product was bound to Streptavidin Sepharose HRP beads (Cytiva, Marlborough, MA, USA) via agitation for 10 min at 1500 rpm. A handheld vacuum plate (QIAvac 24, Qiagen) was used to isolate biotinylated single-strand DNA from the bead-amplicon. This strand was annealed (80 °C for two minutes) to the sequencing primer 5′-GGGGGTTTTGGTTGT. Pyrosequencing was performed on the PyroMark Q96 system using Q96 cartridges (Qiagen). The percentage methylation of each CpG site within the amplicon was calculated using C/[C + T].

### 4.5. ChIP-qPCR

Tissue samples were cut into 1 mm pieces and treated using the EpiQuik Tissue ChIP Kit (P-2003-2, Epigentek, Farmingdale, NY, USA), fixed using 1% formaldehyde at RT for 5 min, and washed in ice-cold 1× PBS prior to Dounce homogenisation and centrifugation at 5000 rpm for 5 min. Chromatin was prepared from the pellet, re-suspended in lysis buffer with protease inhibitors, and sonicated using a Diagenode Bioruptor Plus (Denvile, NJ, USA), for 20 × 30 s on/30 s off. To clear cell debris, sonicates were centrifuged at 14,000 rpm for 10 min at 4 °C. Chromatin was diluted and ChIP performed according to the manufacturer’s instructions using antibodies (1 μg/μL) against RNA polymerase II (positive control), mouse antibody immunoglobulin G (IgG) (negative control), H3K27ac (ab4729, Abcam), or H3K4me3 (ab8580, Abcam). Five-percent input samples, not subject to any antibody treatment, were run in parallel for quantification analysis. Decrosslinking was performed following immunoprecipitation and proteins treated by proteinase K. DNA was eluted and purified using the Qiagen QIAquick PCR Purification Kit. qPCR was performed as described above using primers targeting the promoter region of *Gapdh* (positive control) F: 5′-TCTCTGCTCCTCCCTGTTCT, R: 5′-TACGGCCAAATCCGTTCACA, *Sat2* (negative control) F: 5′-ACAGCTACTGGAAACGGCTGA, R: 5′-CTCAGGGCTTCTTCACTGATCT), *Nr3c1 Exon 1_7_* F: 5′-CTGTAGCCCCTCTGCTAGT, R: 5′-TAGTTTCTCTTCTCCCAGGC; *Nr3c1 (−1 kb TSS)* F: 5′-AAGGGTTAGAAGGAATTTGGGGA, R: 5′-TGACGTGCCAGAGCCAATTA:. ChIP-qPCR data were expressed as the percentage of input chromatin.

### 4.6. Behavioural Measures of Anxiety

Behavioural testing was conducted between 10:00 and 16:00, with a pseudo-random distribution of testing for rats of different genotypes. Rats were habituated to the test rooms for 30 min prior to testing. Rats were tested individually, and the apparatus was cleaned with a 70% ethanol between subjects. Testing in the open field (OF) occurred 3 days before testing in the elevated plus maze (EPM). Both tests are widely used paradigms for assessing innate anxiety indexed by the competition between the exploration of novel contexts and the aversion to open, brightly lit environments or heights [86,87,88,89].

The OF (opaque black plastic box 100 cm × 100 cm × 40 cm high) was illuminated at 30–35 lx. Animals were placed into the centre of the box and their location monitored for 10 min. The EPM consisted of two open arms and two closed arms (50 cm × 10 cm black wood floor, 10 cm black plexiglass walls surrounded by closed arms, equivalent arms arranged oppositionally) elevated by 70 cm under low (30–35 lx) light surrounded by a dark-blue floor–ceiling curtain to eliminate external context stimulation. Animals were placed in the centre of the EPM facing an open arm and their position recorded for 5 min. Data were collected using EthoVision XT 13 software (Noldus Information Technology, Wageningen, The Netherlands). Each rat was tracked (12 frames/s) for location of its greater body proportion for time spent in a virtual 50% area central zone and zone closest to the arena walls (OF), or total time spent in the open arm (EPM).

### 4.7. Statistics

All data were analysed using RStudio v2024.04.1. Outcome measures by genotype were analysed using *t*-tests. Where both sexes were included, and therefore both genotype and sex were included in the analysis, this was carried out using linear regression. Initially all models included sex, genotype and the sex-by-genotype interaction. AIC score was then used to determine whether model fit improved upon the removal of the interaction term. Two-sided tests were used apart from instances in which results were replicated in a separate cohort with an a priori directional hypothesis. Analysis using linear models such as ANOVA and linear regression rely on the assumption of normality and homogeneity of variance. We checked the model residuals for normality and equal variance. In most cases, the residuals were not normally distributed, and heterogeneity of variance was observed. In addition, the basis of the heterogeneity differed between experimental measures. The appropriate transformation to achieve homogeneity of variances in each case was chosen using the ‘boxcox’ function from the R package ‘MASS 7.3-65’ and applied to the dependent variable before running the regression model.

## Data Availability

Data that support the findings of this study are available from the corresponding author upon reasonable request, subject to ethical and privacy considerations.

## Supplementary Materials

The following supporting information can be downloaded at https://www.mdpi.com/article/10.3390/ijms26125547/s1.

## Abbreviations

The following abbreviations are used in this manuscript:

CBG	Corticosteroid-binding globulin
CORT	Cortisol/corticosterone
CRH	Corticotrophin-releasing hormone
EPM	Elevated plus maze
GR	Glucocorticoid receptor
HET	Heterozygous *Cacna1c* rat
HPA	Hypothalamus–pituitary–adrenal
L-VGCC	L-type voltage-gated calcium channel
MR	Mineralocorticoid receptor
OF	Open field
PFC	Prefrontal cortex
PTSD	Post-traumatic stress disorder
SNP	Single-nucleotide polymorphism

## References

[1] Ferreira M.A.R., O’Donovan M.C., Meng Y.A., Jones I.R., Ruderfer D.M., Jones L., Fan J., Kirov G., Perlis R.H., Green E.K. (2008). Collaborative genome-wide association analysis supports a role for *ANK3* and *CACNA1C* in bipolar disorder. Nat. Genet..

[2] Green E.K., Grozeva D., Jones I., Jones L., Kirov G., Caesar S., Gordon-Smith K., Fraser C., Forty L., Russell E. (2010). The bipolar disorder risk allele at *CACNA1C* also confers risk of recurrent major depression and of schizophrenia. Mol. Psychiatry.

[3] Zheng F., Zhang Y., Xie W., Li W., Jin C., Mi W., Wang F., Ma W., Ma C., Yang Y. (2014). Further evidence for genetic association of *CACNA1C* and schizophrenia: New risk loci in a Han Chinese population and a meta-analysis. Schizophr. Res..

[4] Takahashi S., Glatt S.J., Uchiyama M., Faraone S.V., Tsuang M.T. (2015). Meta-analysis of data from the Psychiatric Genomics Consortium and additional samples supports association of *CACNA1C* with risk for schizophrenia. Schizophr. Res..

[5] Bigos K.L., Mattay V.S., Callicott J.H., Straub R.E., Vakkalanka R., Kolachana B., Hyde T.M., Lipska B.K., Kleinman J.E., Weinberger D.R. (2010). Genetic Variation in *CACNA1C* Affects Brain Circuitries Related to Mental Illness. Arch. Gen. Psychiatry.

[6] Roussos P., Mitchell A.C., Voloudakis G., Fullard J.F., Pothula V.M., Tsang J., Stahl E.A., Georgakopoulos A., Ruderfer D.M., Charney A. (2014). A Role for Noncoding Variation in Schizophrenia. Cell Rep..

[7] Starnawska A., Demontis D., Pen A., Hedemand A., Nielsen A.L., Staunstrup N.H., Grove J., Als T.D., Jarram A.,  O’Brien N.L. (2016). *CACNA1C* hypermethylation is associated with bipolar disorder. Transl. Psychiatry.

[8] Eckart N., Song Q., Yang R., Wang R., Zhu H., McCallion A.S., Avramopoulos D. (2016). Functional Characterization of Schizophrenia-Associated Variation in *CACNA1C*. PLoS ONE.

[9] Yoshimizu T., Pan J.Q., Mungenast A.E., Madison J.M., Su S., Ketterman J., Ongur D., Mcphie D., Cohen B., Perlis R. (2015). Functional implications of a psychiatric risk variant within *CACNA1C* in induced human neurons. Mol. Psychiatry.

[10] Jaffe A.E., Hoeppner D.J., Saito T., Blanpain L., Ukaigwe J., Burke E.E., Collado-Torres L., Tao R., Tajinda K., Maynard K.R. (2020). Profiling gene expression in the human dentate gyrus granule cell layer reveals insights into schizophrenia and its genetic risk. Nat. Neurosci..

[11] Berger S.M., Bartsch D. (2014). The role of L-type voltage-gated calcium channels Cav1.2 and Cav1.3 in normal and pathological brain function. Cell Tissue Res..

[12] Vierra N.C., O’Dwyer S.C., Matsumoto C., Fernando Santana L., Trimmer J.S. (2021). Regulation of neuronal excitation-transcription coupling by Kv2.1-induced clustering of somatic L-type Ca^2+^ channels at ER-PM junctions. Proc. Natl. Acad. Sci. USA.

[13] Trubetskoy V., Pardiñas A.F., Qi T., Panagiotaropoulou G., Awasthi S., Bigdeli T.B., Bryois J., Chen C.Y., Dennison C.A., Hall L.S. (2022). Mapping genomic loci implicates genes and synaptic biology in schizophrenia. Nature.

[14] Murri M.B., Fanelli F., Pagotto U., Bonora E., Triolo F., Chiri L., Allegri F., Mezzullo M., Menchetti M., Mondelli V. (2016). Neuroactive Steroids in First-Episode Psychosis: A Role for Progesterone?. Schizophr. Res. Treat..

[15] Heim C., Newport D.J., Mletzko T., Miller A.H., Nemeroff C.B. (2008). The link between childhood trauma and depression: Insights from HPA axis studies in humans. Psychoneuroendocrinology.

[16] MCEWEN Harold B.S., Milliken M. (2004). Protection and Damage from Acute and Chronic Stress: Allostasis and Allostatic Overload and Relevance to the Pathophysiology of Psychiatric Disorders. Ann. N. Y. Acad. Sci..

[17] Phillips L.J., Mcgorry P.D., Garner B., Thompson K.N., Pantelis C., Wood S.J., Berger G. (2006). Stress, the Hippocampus and the Hypothalamic-Pituitary-Adrenal Axis: Implications for the Development of Psychotic Disorders. Aust. N. Z. J. Psychiatry.

[18] Zorn J.V., Schür R.R., Boks M.P., Kahn R.S., Joëls M., Vinkers C.H. (2017). Cortisol stress reactivity across psychiatric disorders: A systematic review and meta-analysis. Psychoneuroendocrinology.

[19] Dedic N., Pöhlmann M.L., Richter J.S., Mehta D., Czamara D., Metzger M.W., Dine J., Bedenk B.T., Hartmann J., Wagner K.V. (2018). Cross-disorder risk gene *CACNA1C* differentially modulates susceptibility to psychiatric disorders during development and adulthood. Mol. Psychiatry.

[20] Zhao M., Yang J., Qiu X., Yang X., Qiao Z., Song X., Wang L., Zhao E., Yang Y., Cao D. (2020). *CACNA1C* rs1006737, Threatening Life Events, and Gene–Environment Interaction Predict Major Depressive Disorder. Front. Psychiatry.

[21] Bastos C.R., Tovo-Rodrigues L., Ardais A.P., Xavier J., Salerno P.S.V., Camerini L., Jansen K., de Mattos Souza L.D., da Silva R.A., Lara D.R. (2020). The role of *CACNA1C* gene and childhood trauma interaction on bipolar disorder. Prog. Neuropsychopharmacol. Biol. Psychiatry.

[22] Klaus K., Butler K., Gutierrez H., Durrant S.J., Pennington K. (2018). Interactive effects of early life stress and *CACNA1C* genotype on cortisol awakening response. Biol. Psychol..

[23] Terrillion C.E., Francis T.C., Puche A.C., Lobo M.K., Gould T.D. (2017). Decreased Nucleus Accumbens Expression of Psychiatric Disorder Risk Gene *Cacna1c* Promotes Susceptibility to Social Stress. Int. J. Neuropsychopharmacol..

[24] Ehlinger D.G., Commons K.G. (2019). Cav1.2 L-type calcium channels regulate stress coping behavior via serotonin neurons. Neuropharmacology.

[25] Karst H., Nair S., Velzing E., Rumpff-van Essen L., Slagter E., Shinnick-Gallagher P., Joëls M. (2002). Glucocorticoids alter calcium conductances and calcium channel subunit expression in basolateral amygdala neurons. Eur. J. Neurosci..

[26] Joëls M., Velzing E., Nair S., Verkuyl J.M., Karst H. (2003). Acute stress increases calcium current amplitude in rat hippocampus: Temporal changes in physiology and gene expression. Eur. J. Neurosci..

[27] Qin Y., Karst H., Joëls M. (2004). Chronic unpredictable stress alters gene expression in rat single dentate granule cells. J. Neurochem..

[28] Van Gemert N.G., Joëls M. (2006). Effect of Chronic Stress and Mifepristone Treatment on Voltage-Dependent Ca^2+^ Currents in Rat Hippocampal Dentate Gyrus. J. Neuroendocr..

[29] Maigaard K., Hageman I., Jørgensen A., Jørgensen M.B., Wörtwein G. (2012). Electroconvulsive stimulations prevent chronic stress-induced increases in L-type calcium channel mRNAs in the hippocampus and basolateral amygdala. Neurosci. Lett..

[30] Jaric I., Rocks D., Cham H., Herchek A., Kundakovic M. (2019). Sex and estrous cycle effects on anxiety- and depression-related phenotypes in a two-hit developmental stress model. Front. Mol. Neurosci..

[31] Bavley C.C., Fischer D.K., Rizzo B.K., Rajadhyaksha A.M. (2017). Cav1.2 channels mediate persistent chronic stress-induced behavioral deficits that are associated with prefrontal cortex activation of the p25/Cdk5-glucocorticoid receptor pathway. Neurobiol. Stress..

[32] Jacobson L. (2005). Hypothalamic–Pituitary–Adrenocortical Axis Regulation. Endocrinol. Metab. Clin..

[33] de Kloet E.R., de Kloet S.F., de Kloet C.S., de Kloet A.D. (2019). Top-down and bottom-up control of stress-coping. J. Neuroendocrinol..

[34] Gjerstad J.K., Lightman S.L., Spiga F. (2018). Role of glucocorticoid negative feedback in the regulation of HPA axis pulsatility. Stress.

[35] Lamers F., Vogelzangs N., Merikangas K.R., De Jonge P., Beekman A.T.F., Penninx B.W.J.H. (2012). Evidence for a differential role of HPA axis function, inflammation and metabolic syndrome in melancholic versus atypical depression. Mol. Psychiatry.

[36] Silverman M.N., Sternberg E.M. (2012). Glucocorticoid regulation of inflammation and its functional correlates: From HPA axis to glucocorticoid receptor dysfunction. Ann. N. Y. Acad. Sci..

[37] Dallman M.F., Hellhammer D., Contrada R., Baum A. (2011). Regulation of the hypothalamo-pituitaryadrenal axis, chronic stress, and energy: The role of brain networks. The Handbook of Stress Science: Biology, Psychology, and Health.

[38] Harris A.P., Holmes M.C., de Kloet E.R., Chapman K.E., Seckl J.R. (2013). Mineralocorticoid and glucocorticoid receptor balance in control of HPA axis and behaviour. Psychoneuroendocrinology.

[39] de Kloet E.R., Otte C., Kumsta R., Kok L., Hillegers M.H.J., Hasselmann H., Kliegel D., Joëls M. (2016). Stress and Depression: A Crucial Role of the Mineralocorticoid Receptor. J. Neuroendocr..

[40] Ratka A., Sutanto W., Bloemers M., de Kloet R. (1989). On the Role of Brain Mineralocorticoid (Type I) and Glucocorticoid (Type II) Receptors in Neuroendocrine Regulation. Neuroendocrinology.

[41] McEwen B.S. (2008). Central effects of stress hormones in health and disease: Understanding the protective and damaging effects of stress and stress mediators. Eur. J. Pharmacol..

[42] Chrousos G.P., Kino T. (2009). Glucocorticoid Signaling in the Cell. Ann. N. Y. Acad. Sci..

[43] Heim C., Ehlert U., Hellhammer D.H. (2000). The potential role of hypocortisolism in the pathophysiology of stress-related bodily disorders. Psychoneuroendocrinology.

[44] Raison C.L., Miller A.H. (2003). When Not Enough Is Too Much: The Role of Insufficient Glucocorticoid Signaling in the Pathophysiology of Stress-Related Disorders. Am. J. Psychiatry.

[45] Juruena M.F., Eror F., Cleare A.J., Young A.H. (2020). The Role of Early Life Stress in HPA Axis and Anxiety. Anxiety Disord..

[46] Weaver I.C.G., D’Alessio A.C., Brown S.E., Hellstrom I.C., Dymov S., Sharma S., Szyf M., Meaney M.J. (2007). The Transcription Factor Nerve Growth Factor-Inducible Protein A Mediates Epigenetic Programming: Altering Epigenetic Marks by Immediate-Early Genes. J. Neurosci..

[47] Weaver I.C.G., Diorio J., Seckl J.R., Szyf M., Meaney M.J. (2004). Early Environmental Regulation of Hippocampal Glucocorticoid Receptor Gene Expression: Characterization of Intracellular Mediators and Potential Genomic Target Sites. Ann. N. Y. Acad. Sci..

[48] Palma-Gudiel H., Córdova-Palomera A., Leza J.C., Fañanás L. (2015). Glucocorticoid receptor gene (NR3C1) methylation processes as mediators of early adversity in stress-related disorders causality: A critical review. Neurosci. Biobehav. Rev..

[49] Weaver I.C.G., Cervoni N., Champagne F.A., D’Alessio A.C., Sharma S., Seckl J.R., Dymov S., Szyf M., Meaney M.J. (2004). Epigenetic programming by maternal behavior. Nat. Neurosci..

[50] Seo M.K., Kim S., Seog D.-H., Bahk W.-M., Kim S.-H., Park S.W., Lee J.G. (2020). Effects of Early Life Stress on Epigenetic Changes of the Glucocorticoid Receptor 17 Promoter during Adulthood. Int. J. Mol. Sci..

[51] Miller J.L., Grant P.A. (2013). The role of DNA methylation and histone modifications in transcriptional regulation in humans. Subcell. Biochem..

[52] Creyghton M.P., Cheng A.W., Welstead G.G., Kooistra T., Carey B.W., Steine E.J., Hanna J., Lodato M.A., Frampton G.M., Sharp P.A. (2010). Histone H3K27ac separates active from poised enhancers and predicts developmental state. Proc. Natl. Acad. Sci. USA.

[53] Herman J.P. (2022). The neuroendocrinology of stress: Glucocorticoid signaling mechanisms. Psychoneuroendocrinology.

[54] Meyer E.J., Nenke M.A., Rankin W., Lewis J.G., Torpy D.J. (2016). Corticosteroid-Binding Globulin: A Review of Basic and Clinical Advances. Horm. Metab. Res..

[55] McCormick J.A., Lyons V., Jacobson M.D., Noble J., Diorio J., Nyirenda M., Weaver S., Ester W., Yau J.L.W., Meaney M.J. (2000). 5′-Heterogeneity of Glucocorticoid Receptor Messenger RNA Is Tissue Specific: Differential Regulation of Variant Transcripts by Early-Life Events. Mol. Endocrinol..

[56] McEwen B.S. (2000). Protective and damaging effects of stress mediators: Central role of the brain. Progress in Brain Research.

[57] McEwen B.S., Akil H. (2020). Revisiting the Stress Concept: Implications for Affective Disorders. J. Neurosci..

[58] Agorastos A., Chrousos G.P. (2022). The neuroendocrinology of stress: The stress-related continuum of chronic disease development. Mol. Psychiatry.

[59] Lee A.S., Ra S., Rajadhyaksha A.M., Britt J.K., De Jesus-Cortes H., Gonzales K.L., Lee A., Moosmang S., Hofmann F., Pieper A.A. (2012). Forebrain elimination of *cacna1c* mediates anxiety-like behavior in mice. Mol. Psychiatry.

[60] Dao D.T., Mahon P.B., Cai X., Kovacsics C.E., Blackwell R.A., Arad M., Shi J., Zandi P.P., O’Donnell P., Knowles J.A. (2010). Mood Disorder Susceptibility Gene *CACNA1C* Modifies Mood-Related Behaviors in Mice and Interacts with Sex to Influence Behavior in Mice and Diagnosis in Humans. Biol. Psychiatry.

[61] Harro J. (2018). Animals, anxiety, and anxiety disorders: How to measure anxiety in rodents and why. Behav. Brain Res..

[62] Dam H., Buch J.O.D., Nielsen A.B., Weikop P., Jørgensen M.B. (2022). The association of anxiety and other clinical features with *CACNA1C* rs1006737 in patients with depression. Transl. Neurosci..

[63] Kabir Z.D., Che A., Fischer D.K., Rice R.C., Rizzo B.K., Byrne M., Glass M.J., De Marco Garcia N.V., Rajadhyaksha A.M. (2017). Rescue of impaired sociability and anxiety-like behavior in adult *cacna1c*-deficient mice by pharmacologically targeting eIF2α. Mol. Psychiatry.

[64] Temme S.J., Murphy G.G. (2017). The L-type voltage-gated calcium channel Ca _V_ 1.2 mediates fear extinction and modulates synaptic tone in the lateral amygdala. Learn. Mem..

[65] Klomp A.J., Plumb A., Mehr J.B., Madencioglu D.A., Wen H., Williams A.J. (2022). Neuronal deletion of CaV1.2 is associated with sex-specific behavioral phenotypes in mice. Sci. Rep..

[66] Sykes L., Haddon J., Lancaster T.M., Sykes A., Azzouni K., Ihssen N., Moon A.L., Lin T.C.E., Linden D.E., Owen M.J. (2019). Genetic Variation in the Psychiatric Risk Gene *CACNA1C* Modulates Reversal Learning Across Species. Schizophr. Bull..

[67] Mitra R., Sapolsky R.M. (2008). Acute corticosterone treatment is sufficient to induce anxiety and amygdaloid dendritic hypertrophy. Proc. Natl. Acad. Sci. USA.

[68] Murray F., Smith D.W., Hutson P.H. (2008). Chronic low dose corticosterone exposure decreased hippocampal cell proliferation, volume and induced anxiety and depression like behaviours in mice. Eur. J. Pharmacol..

[69] Kim H., Yi J.H., Choi K., Hong S., Shin K.S., Kang S.J. (2014). Regional differences in acute corticosterone-induced dendritic remodeling in the rat brain and their behavioral consequences. BMC Neurosci..

[70] Novaes L.S., dos Santos N.B., Perfetto J.G., Goosens K.A., Munhoz C.D. (2018). Environmental enrichment prevents acute restraint stress-induced anxiety-related behavior but not changes in basolateral amygdala spine density. Psychoneuroendocrinology.

[71] Smedler E., Louhivuori L., Romanov R.A., Masini D., Dehnisch Ellström I., Wang C., Caramia M., West Z., Zhang S., Rebellato P. (2022). Disrupted *Cacna1c* gene expression perturbs spontaneous Ca^2+^ activity causing abnormal brain development and increased anxiety. Proc. Natl. Acad. Sci. USA.

[72] Boyle M.P., Brewer J.A., Funatsu M., Wozniak D.F., Tsien J.Z., Izumi Y., Muglia L.J. (2005). Acquired deficit of forebrain glucocorticoid receptor produces depression-like changes in adrenal axis regulation and behavior. Proc. Natl. Acad. Sci. USA.

[73] Bitran D., Shiekh M., Dowd J.A., Dugan M.M., Renda P. (1998). Corticosterone Is Permissive to the Anxiolytic Effect That Results From the Blockade of Hippocampal Mineralocorticoid Receptors. Pharmacol. Biochem. Behav..

[74] Handa R.J., Nunley K.M., Lorens S.A., Louie J.P., McGivern R.F., Bollnow M.R. (1994). Androgen regulation of adrenocorticotropin and corticosterone secretion in the male rat following novelty and foot shock stressors. Physiol. Behav..

[75] Rhodes M.E., Rubin R.T. (1999). Functional sex differences (`sexual diergism’) of central nervous system cholinergic systems, vasopressin, and hypothalamic–pituitary–adrenal axis activity in mammals: A selective review. Brain Res. Rev..

[76] Droste S.K., De Groote L., Lightman S.L., Reul J.M.H.M., Linthorst A.C.E. (2009). The Ultradian and Circadian Rhythms of Free Corticosterone in the Brain are Not Affected by Gender: An In Vivo Microdialysis Study in Wistar Rats. J. Neuroendocr..

[77] Solomon M.B., Loftspring M., de Kloet A.D., Ghosal S., Jankord R., Flak J.N., Wulsin A.C., Krause E.G., Zhang R., Rice T. (2015). Neuroendocrine Function After Hypothalamic Depletion of Glucocorticoid Receptors in Male and Female Mice. Endocrinology.

[78] Minni A.M., de Medeiros G.F., Helbling J.C., Duittoz A., Marissal-Arvy N., Foury A., De Smedt-Peyrusse V., Pallet V., Moisan M.P. (2014). Role of corticosteroid binding globulin in emotional reactivity sex differences in mice. Psychoneuroendocrinology.

[79] Heck A.L., Handa R.J. (2019). Sex differences in the hypothalamic–pituitary–adrenal axis’ response to stress: An important role for gonadal hormones. Neuropsychopharmacology.

[80] Pennington K., Klaus K., Fachim H.A., Butler K., Trischel K., Dalton C.F., Heald A., Reynolds G.P. (2020). *CACNA1C* Methylation: Association with Cortisol, Perceived Stress, rs1006737 and Childhood Trauma in Males. Epigenomics.

[81] Bali A., Gupta S., Singh N., Jaggi A.S. (2013). Implicating the role of plasma membrane localized calcium channels and exchangers in stress-induced deleterious effects. Eur. J. Pharmacol..

[82] Tigaret C.M., Lin T.-C.E., Morrell E.R., Sykes L., Moon A.L., O’Donovan M.C., Owen M.J., Wilkinson L.S., Jones M.W., Thomas K.L. (2021). Neurotrophin receptor activation rescues cognitive and synaptic abnormalities caused by hemizygosity of the psychiatric risk gene *Cacna1c*. Mol. Psychiatry.

[83] de Kloet E.R., Joëls M. (2024). The cortisol switch between vulnerability and resilience. Mol. Psychiatry.

[84] Hodes G.E., Epperson C.N. (2019). Sex Differences in Vulnerability and Resilience to Stress Across the Life Span. Biol. Psychiatry.

[85] Gobinath A.R., Choleris E., Galea L.A.M. (2017). Sex, hormones, and genotype interact to influence psychiatric disease, treatment, and behavioral research. J. Neurosci. Res..

[86] Kisko T.M., Braun M.D., Michels S., Witt S.H., Rietschel M., Culmsee C., Schwarting R.K.W., Wöhr M. (2018). *Cacna1c* haploinsufficiency leads to pro-social 50-kHz ultrasonic communication deficits in rats. DMM Dis. Models Mech..

[87] Denenberg V.H. (1969). Open-Field Behavior in the Rat: What Does it Mean?. Ann. N. Y. Acad. Sci..

[88] Pellow S., Chopin P., File S.E., Briley M. (1985). Validation of open: Closed arm entries in an elevated plus-maze as a measure of anxiety in the rat. J. Neurosci. Methods.

[89] Walf A.A., Frye C.A. (2007). The use of the elevated plus maze as an assay of anxiety-related behavior in rodents. Nat. Protoc..

