# Supplementary material for: Reduced Gene Dosage of the Psychiatric Risk Gene Cacna1c Is Associated with Impairments in Hypothalamic–Pituitary–Adrenal Axis Activity in Rats
Source: Int J Mol Sci. 2025 Jun 10;26(12):5547. doi: 10.3390/ijms26125547 (PMC12192671; doi:10.3390/ijms26125547)
Supplement: Supplementary file 1 [file ijms-26-05547-s001.zip › ijms-3597904-supplementary.pdf]

International Journal of
Molecular Sciences

MDPI

Supplementary Materials

Figure S1. Figure 1 with transformations corresponding to those used in the analysis.

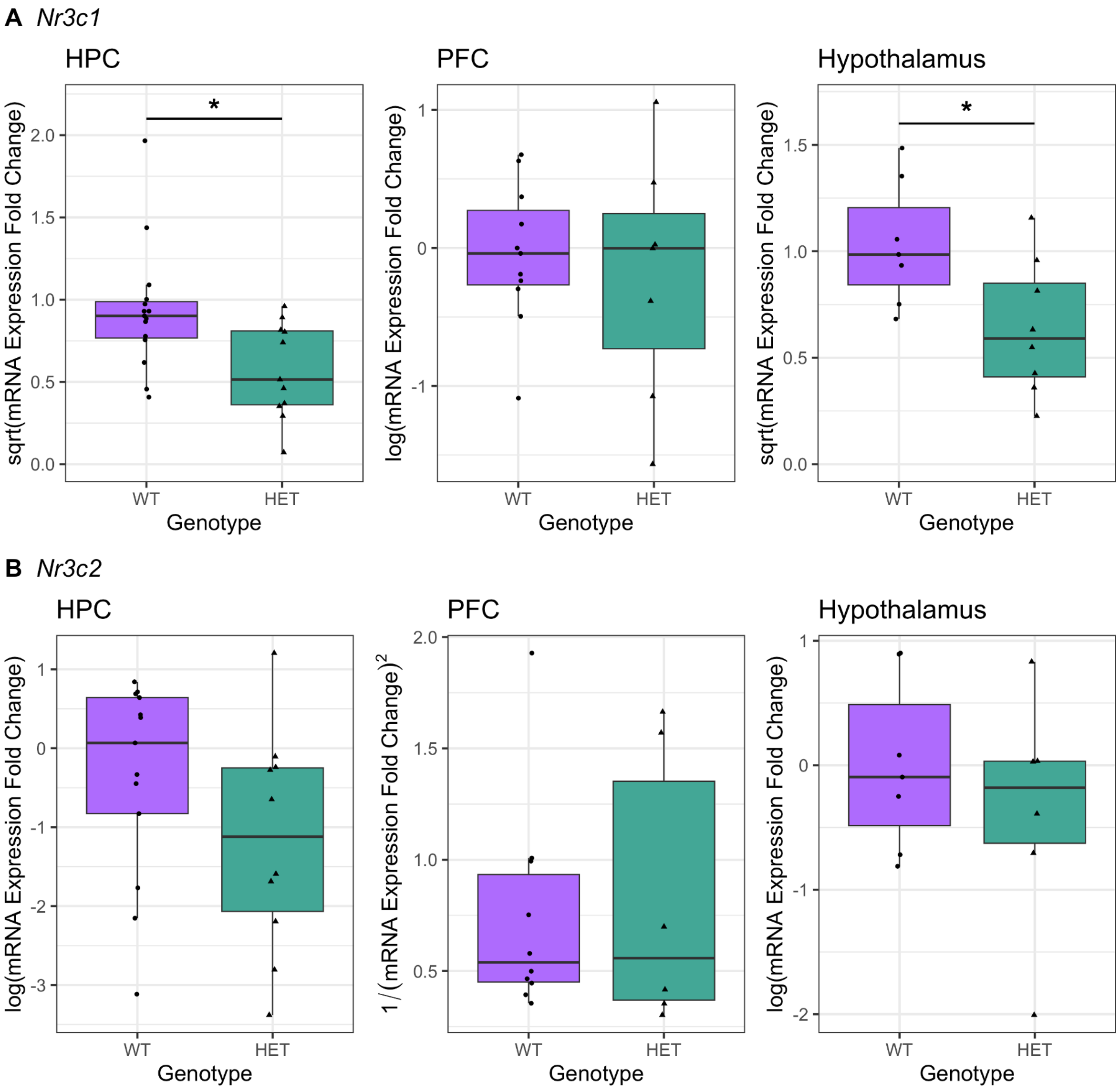

Figure S1. (a). *Cacna1c*$^{+/-}$ rats (HET) have reduced *Nr3c1* gene expression in the hippocampus and the hypothalamus compared to wild-types (WT). (b). There are no differences in *Nr3c2* expression in any brain region tested. Medians and quartiles are depicted on each plot (whiskers run to the smallest datapoint within 1.5 x IQR below Q1 and the largest datapoint within 1.5IQR above Q3). Measures from individual rats are shown as black dots. Results were considered significant if $p < 0.05$ (*). Dependent variables are presented corresponding to the transformation used in the analysis *Nr3c1*: Hippocampus, HET n = 11, WT n = 15; PFC, HET n = 7, WT n = 11; Hypo HET n = 8, WT n = 7. *Nr3c2*: Hippocampus, HET n = 10, WT n = 13; PFC, HET n = 6, WT n = 10; Hypo HET n = 6, WT n = 7.

Figure S2. Figure 2 with transformations corresponding to those used in the analysis.

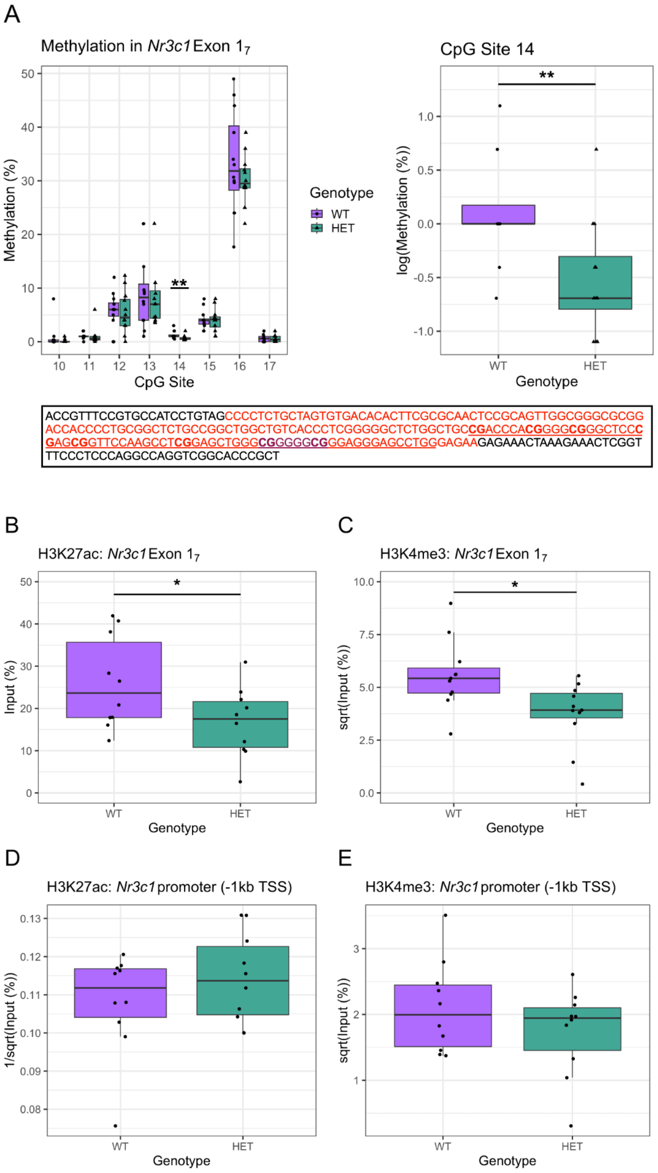

Figure S2. (a) *Top*: The % of methylation at each targeted CpG site in the hippocampus of WT and *Cacna1c*$^{+/-}$ rats is displayed (*Top Left*) with no significant differences between the genotypes, with the exception of CpG 14 (*Top Right*). The highest % methylation was within at the 5′ CpG site (CpG$_{16}$) within the *NGFIA* binding site. *Bottom*: Schematic showing the genetic sequence that indicates Exon 1$_7$ within the promoter region of *Nr3c1* in the rat. The red region indicates the sequence of Exon 1$_7$, with the *NGFIA* binding site contained within, highlighted in purple. The sequence analysed by this study is underlined and the CpG dinucleotides investigated are in bold (corresponding to CpGs 10-17. n = 12 per group. (**b-e**) *Cacna1c*$^{+/-}$ rats show reduced DNA interacting with histone modification markers of active transcription H3K4me3 and H3K27ac within the exon 1$_7$ region (**b, c**). No differences were seen in the region closer to the transcription start site (**d, e**). Medians and quartiles are depicted on each plot (whiskers run to the smallest datapoint within 1.5 x IQR below Q1 and the largest datapoint within 1.5IQR above Q3). n = 10 per group per group except c where n = 11. Measures from individual rats are shown as black dots. Dependent variables were presented corresponding to the transformation used in the analysis. Results were considered significant if $p < 0.05$ (*) or $p < 0.05$ (*).

Figure S3. *Nr3c1* Exon 1_7 expression in the hippocampus of WT and HETs

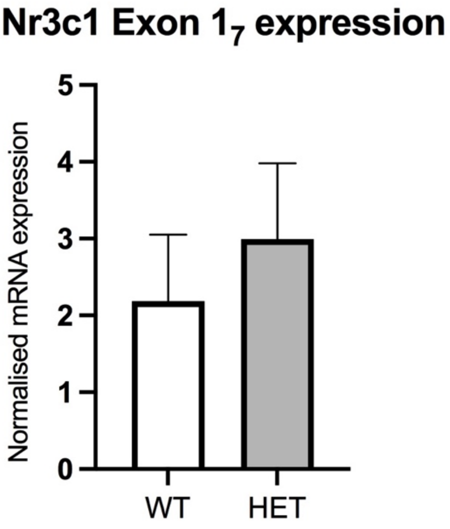

Figure S3. No differences were seen in the expression *of Nr3c1 exon* 1_7 in the hippocampus between wild-type (WT) and *Cacna1c* hemizygous rats (HET) (p = 0.555, n = 8 per group) as measured by qPCR. Expression levels were normalised *Gapdh* and *Hprt* expression. Data are shown as mean ± SE.

Figure S4. Figure 3 with transformations corresponding to those used in the analysis.

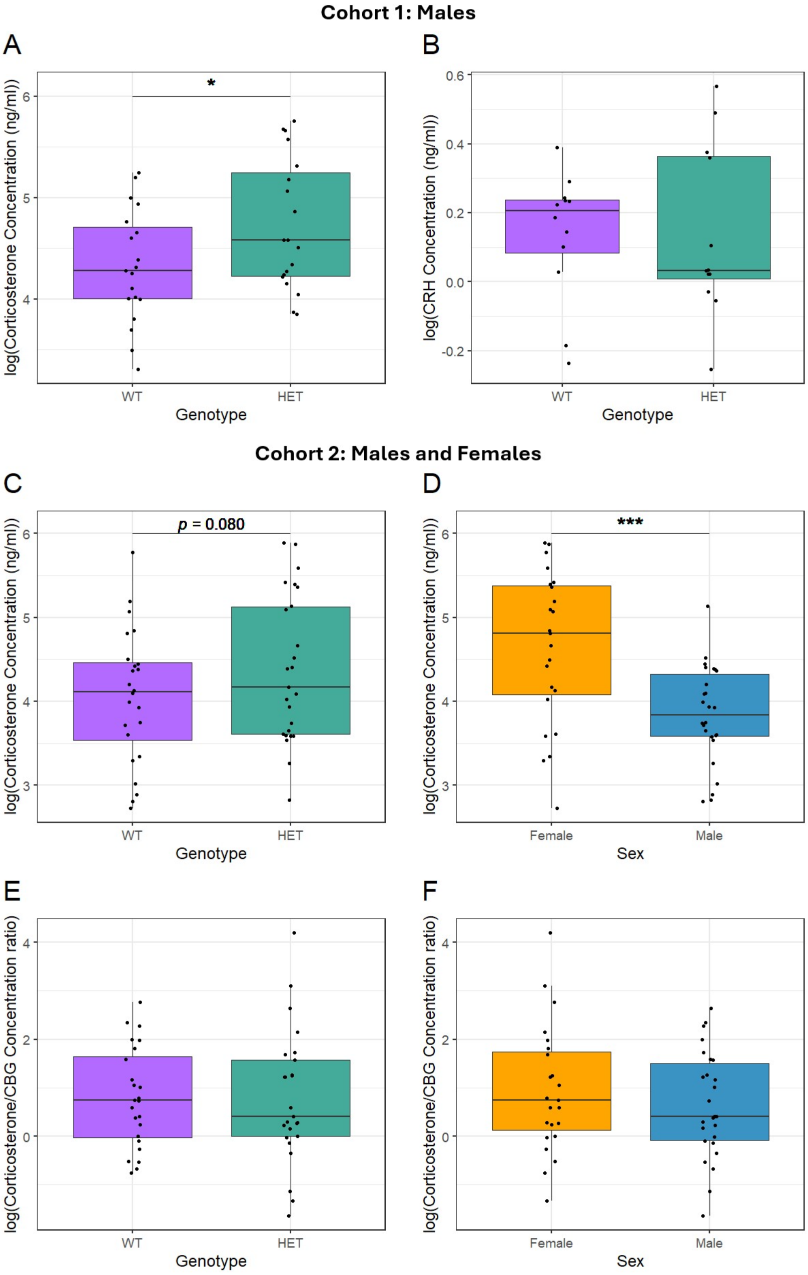

Figure S4 (a) Male *Cacna1c*+/- rats (HET) have higher circulating corticosterone levels than WT males (n = 19 per group). (**b**) Male *Cacna1c*+/- rats have similar CRH hormone levels to male WT (n = 12 per group). In a separate mixed sex cohort (n: Male WT = 13, Male HET = 13, Female WT = 11 and Female HET = 12) **(c)** *Cacna1c*+/- rats showed an increased peripheral corticosterone concentration compared to WT. (**d**) A profound sex difference in corticosterone levels was also observed, with females showing higher levels than males. **(e)** There was no difference in the ratio of peripheral corticosterone/CBG between genotypes ($p = 0.248$). **(f)** Corticosterone/CBG did not differ between sexes ($p = 0.199$). There were no sex by genotype interactions ($p > 0.05$, see main text for details) and main effects are presented only. Medians and quartiles are depicted on each plot (whiskers run to the smallest datapoint within 1.5 x IQR below Q1 and the largest datapoint within 1.5IQR above Q3). Measures from individual rats are shown as black dots. Measures from individual rats are shown as black dots. Dependent variables are presented corresponding to the transformation used in the analysis. * $p < 0.05$, *** $p < 0.001$.

Figure S5. Locomotor activity in the OF and EPM in WT and HETs

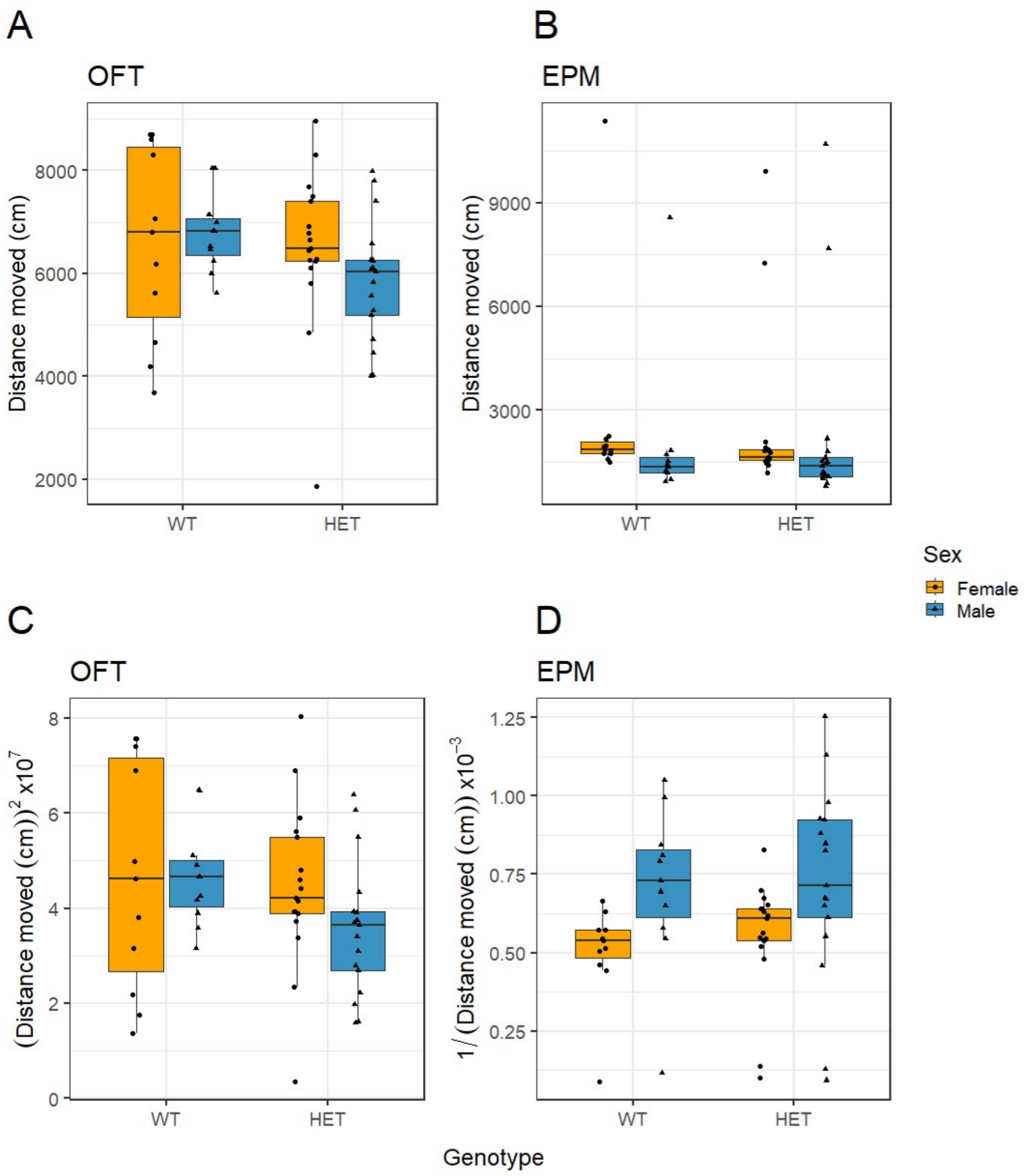

Figure S5. (A). In the OF, there was no effect of *Cacna1c* haploinsufficiency or sex on the locomotor activity as measured by distance travelled in cm (genotype: $t_{(53)} = -1.440$, $p = 0.156$), sex: ($t_{(53)} = 0.248$, $p = 0.805$, genotype x sex: $t_{(53)} = 0.775$, $p = 0.442$). (B.) In the EPM, while there was no effect of *Cacan1c* hemizygosity on activity as measured by total distance travelled in cm ($t_{(53)} = 0.431$, $p = 0.668$),but females travelled more than males ($t_{(53)} = -2.002$, $p = 0.050$). There was no genotype x sex interaction ($t_{(53)} = 0.089$, $p = 0.930$). Untransformed data are presented in A and B, and data transformed in line with analyses are shown in C and D. Measures from individual rats are shown as black dots. Medians and quartiles are depicted on each plot (whiskers run to the smallest datapoint within 1.5 x IQR below Q1 and the largest datapoint within 1.5IQR above Q3). Male HET n = 17; Male WT n = 11; Female HET n = 17; Female WT n = 11.

Figure S6. Figure 4 with transformations corresponding to those used in the analysis.

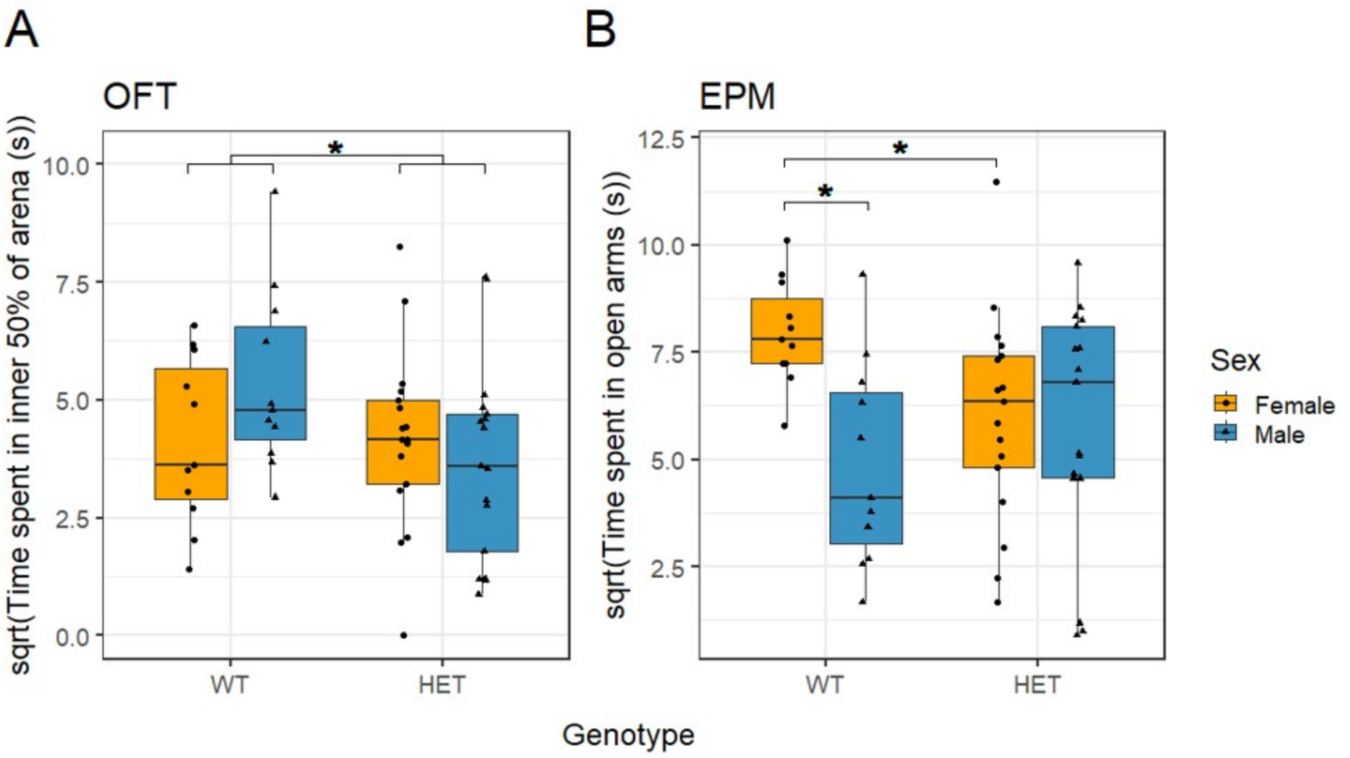

Figure S6. (a) There is a reduction in time spent in the inner 50% of an OF arena in *Cacna1c*$^{+/-}$ rats (HET) compared to WT animals. **(b)** In the same cohort, there were no differences of genotype on the time spent in the open arms of the EPM. However, female rats spent more time in the open arms compared to males and there was a significant interaction between sex and genotype ($t_{(52)}$ = -2.257, p = 0.028), where female *Cacna1c*$^{+/-}$ rats showed a reduced time in open arms compared to female WT ($t_{(26)}$ = -2.457, p = 0.021). Medians and quartiles are depicted on each plot (whiskers run to the smallest datapoint within 1.5 x IQR below Q1 and the largest datapoint within 1.5IQR above Q3). Dependent variables are presented corresponding to the transformation used in the analysis. Measures from individual rats are shown as black dots. * $p < 0.05$. Male HET n = 17; Male WT n = 11; Female HET n = 17; Female WT n = 11.

Table S1: Statistical reports for pyrosequencing

CpG	$t_{(22)}$ value	p value
10	-1.022	0.318
11	-0.029	0.978
12	-0.199	0.844
13	0.024	0.981
14	-3.006	0.007
15	-0.137	0.892
16	-1.015	0.321
17	<0.001	>0.999
